# Effectiveness of traditional band and loop space maintainer vs 3D-printed space maintainer following the loss of primary teeth: a randomized clinical trial

**DOI:** 10.1038/s41598-024-61743-7

**Published:** 2024-06-18

**Authors:** Bhagyashree Thakur, Anuj Bhardwaj, Alexander Maniangat Luke, Dian Agustin Wahjuningrum

**Affiliations:** 1 Division of District Early Intervention Centre, Department of Dentistry, Thane Civil Hospital, Thane, 400601 India; 2 Department of Conservative Dentistry, Faculty of Dental Medicine, Universitas Airlangga, 60132 Surabaya, Indonesia; 3 Department of Conservative Dentistry and Endodontics, College of Dental Sciences & Hospital, Rau, Indore, 453331 India; 4 Department of Clinical Science, College of Dentistry, Ajman University, Ajman P.O. Box 346, Al-Jurf, UAE; 5 Centre of Medical and Bio-Allied Health Sciences Research (CMBAHSR), Ajman University, Ajman P.O. Box 346, Al-Jurf, UAE

**Keywords:** Space maintainers, 3D printing, Gingival index, Patient satisfaction, Early tooth loss, Dental equipment, Paediatric dentistry

## Abstract

This study evaluates the efficacy of 3D-printed band and loop space maintainers (3D-BLSMs) to mitigate concerns caused by early primary tooth loss in children when compared to their conventional equivalents. Over 9 months, 62 participants aged 6 to 12 years participated in a randomized clinical study. This study evaluated their failure rates (de-cementation, debonding, solder breakage, loop breakage, band breakage, and abutment tooth fracture), gingival health, and patient overall satisfaction. Random assignments were made to place the participants in two groups: traditional band and loop space maintainers or the 3D-BLSMs. The findings show that at 9 months, 3D-BLSMs provided significantly higher survival rates (77.4%) than conventional maintainers (51.6%, p < 0.01). Gum inflammation was mild in both groups, highlighting the need for good oral hygiene. In both groups, patient satisfaction exceeded 90%. Although there was some pain at first with 3D-BLSMs, this eventually subsided and aesthetic preferences disappeared. There were no negative consequences noted, and both groups needed ongoing dental treatment. In conclusion, with excellent patient satisfaction in both groups, 3D-printed space maintainers offer greater long-term durability in reducing dental concerns following early primary tooth loss.

## Introduction

The child’s permanent tooth development can be profoundly disrupted by the early loss of primary teeth. Primary teeth have the function of guiding permanent teeth into correct alignment within the jaw or serve as placeholders. However early loss via extraction, trauma, or deterioration might jeopardize this area and create a cascade of responses to possible issues^1^.

Dental crowding is a significant adverse reaction that occurs when permanent teeth do not have adequate space for eruption precisely, perhaps overlapping or misaligning^2^. This malocclusion may have an impact on the bite’s appearance as well as functionality. Further, problems including tooth rotation, tilting or drifting, and even impaction of permanent teeth can result from space loss. Later in adulthood, these problems may end in more intrusive treatments, including braces or extractions^3^.

When it comes to preventative and interceptive orthodontics, space preservation is critical for maintaining the proper alignment and occlusion of permanent teeth after the premature loss of primary teeth. Following the early loss of primary teeth, space maintainers are essentially used for maintaining the space needed for permanent teeth, preventing occlusal alterations and parafunctional habits. Furthermore, the space maintainers can prevent children from experiencing psychological anguish due to early tooth loss while maintaining functional, cosmetic, and arch length concerns^4^. The safe use of biocompatible space maintainers in pediatric dentistry is ensured by their lack of cytotoxic effects on keratinocyte cell lines. Furthermore, fixed space maintainers, which are made of materials like glass fiber-reinforced composite resin, offer noteworthy therapeutic advantages, such as time and money savings, simplicity of use, and visual attractiveness.

Maintaining the longevity and effectiveness of space maintainers requires regular monitoring and the use of ideal oral hygiene measures. A dental expert must do a comprehensive examination before deciding whether or not a patient needs to utilize space maintainers. This evaluation should take into account the child’s age, cooperation, permanent tooth eruption stage, and the position and number of missing teeth. Initial and long-term dental health can be greatly impacted by early intervention and appropriate evaluation, which can enhance teeth alignment, occlusion, and general oral function^5^.

In pediatric dentistry, various space maintainer types are available. To give stability and maintain space until the permanent tooth erupts, fixed space maintainers like the band and loop, crown and loop, lingual arch, and distal shoe are cemented to neighbouring teeth^6^. Partial dentures, flippers, and Hawley appliances are examples of removable space maintainers that offer convenience and little upkeep. Flippers are removable acrylic prosthetics that are generally used for aesthetic reasons, as opposed to partial dentures, which are supported by surrounding teeth and have artificial teeth. Space maintenance and tooth eruption are made easier by adjustable Hawley appliances with clasps and a palatal wire. The professional judgment and specific patient demands will determine which space maintainer is the best^7–9^.

Due to the many drawbacks of band and loop space maintainers (BLSM), numerous studies on the subject have focused on contrasting conventional BLSM with alternative designs. Relevant results show that, in comparison to their crown and loop counterparts, BLSM have a lower mean survival duration and a noticeably higher failure rate, which is mostly caused by documentation^10^. Conventional BLSM has been associated with reduced gingival health, requires building in a laboratory, and is non-functional, which leads to short survival and many sittings. Additionally, it is not able to prevent adjoining teeth from tilting or turning and prone to recurrent cement loss and dislodgments^11^. As a result, research on fiber-reinforced space maintainers as alternatives is being conducted^12^. While wire bending and cement loss are the main causes of appliance failure with BLSMs, several benefits to using fiber-reinforced composite (FRC) space maintainers include better patient acceptability, easier production, and superior aesthetics^13^. Moreover, because of plaque retention and cement deterioration that surround the band, BLSMs may cause pain, mucosal overgrowth, band displacement, ulceration, and gingival hypertrophy^14^. To sum up, conventional BLSMs have several drawbacks that make continuous innovation in the design of juvenile dental appliances necessary to effectively overcome them.

The field of dentistry has been transformed in recent years since the discovery of 3D printing technology. Complex three-dimensional objects can be produced via 3D printing, which is also referred to as additive manufacturing, using digital models. The manufacturing of space maintainers using 3D printing technology allows for exact personalization, improving comfort and performance while also encouraging improved patient compliance. It makes it possible to create space maintainers with intricate designs and other functionalities suited to certain patients’ requirements^15,16^. The optimized process shortens turnaround time and removes processes that need manual labor. Additionally, 3D printing allows for the development of new materials, such as biocompatible and biodegradable ones that will gradually disintegrate once permanent teeth erupt^17^.

Pawar^18^ and Khanna et al.^19^ provided convincing evidence of the effectiveness of 3D-printed space maintainers, paving the route for their widespread use and opening up their potential for enhancing dental treatment. To the best of the authors' knowledge, no clinical trials have been conducted to gauge the effectiveness of 3D-printed space maintainers. As a result, the current study's objective was to contrast the performance and results of traditional band and loop space maintainers and 3D-printed maintainers. Based on current understanding and the prospective benefits of 3D printing technology, it is hypothesized that 3D-printed band and loop space maintainers would produce better results than traditional techniques.

## Methods

### Study design

This study was approved by the Research and Ethical Committee of the College of Dental Sciences and Hospital, Rau (CDSH/524A/2023; dated: 19/01/2023) and conducted based on the Declaration of Helsinki. Written informed consent was obtained from the parents of all participants after complete research procedures were explained. No vulnerable populations were involved. This investigation was registered as a prospective clinical trial by the Clinical Trials Registry of India (https://ctri.nic.in/, Registration No.: CTRI/2023/06/054553; dated: 30/06/2023). This randomized controlled investigation adopted the Consolidated Standards of Reporting Trials (CONSORT) standards to guarantee clear and comprehensive reporting. The parents or legal guardians of the participating children provided their informed consent to participate in this study.

### Sample size estimation

Using the mean and standard deviation, the gingival health status of the abutment tooth at the 9-month mark was used to estimate the sample size and power. We expected a smaller effect size of 0.75 for our study based on a prior study^11^, which revealed an effect size of 1.5*. For the current investigation, it was calculated that a minimum of 31 participants per group were required to reach a power of 0.80 at a significance level of 0.05. A cohort of 31 healthy children aged 4 to 8 years who registered to the outpatient department with extensively damaged single molars on either side demanding extraction or freshly extracted single molars bilaterally in the same arch or opposite arch were taken into consideration.

### Study participants

Participants in the study had to meet the following inclusion criteria. Clinically, they were healthy children aged 4 to 8 years old with grossly mutilated single molars on either side requiring extraction or freshly extracted single molars bilaterally in the same or opposite arch, as well as sound and healthy teeth adjacent to the extraction site and no abnormal dental conditions such as crossbite, open bite, or deep bite. Participants were required to have an erupting tooth bud and at least 1 mm of bone atop the erupting tooth germ with less than one-third of the root developed on radiographs. Only those who met these requirements were considered eligible and enrolled in the study.

Participants with extensively carious teeth close to the created space and those without teeth on the mesial or distal side of the teeth intended for extraction were excluded from this study.

### Randomization and assignment of interventions

**Figure 1:**
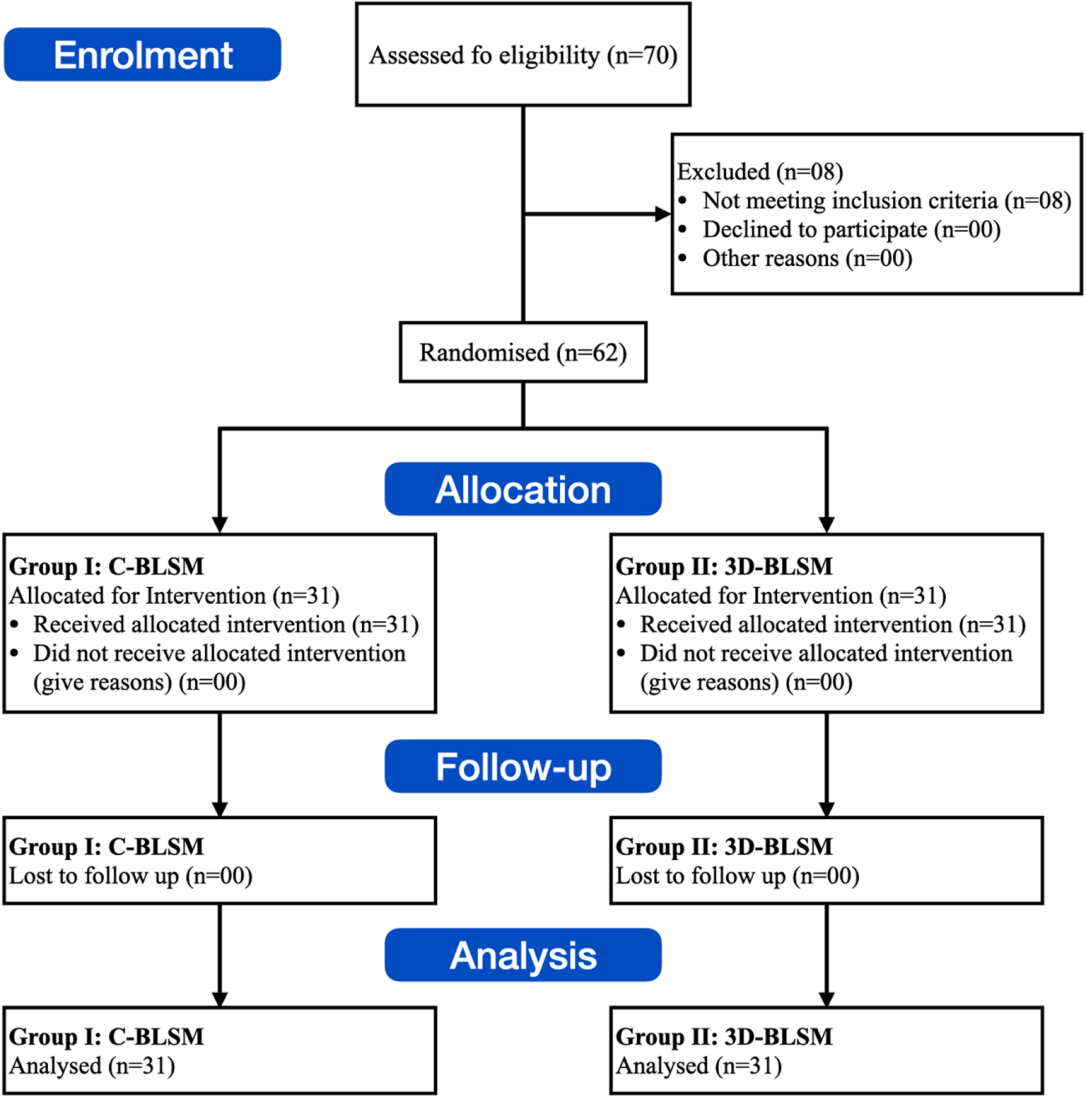
Consort flow diagram for the clinical trial.

For each chosen child, a detailed procedure was performed, beginning with an in-depth medical history and culminating with a thorough clinical examination. Digital intraoral periapical (IOPA) radiographs were taken specifically for the extracted tooth. Impressions were made to produce research models and assess the space that was accessible. The patients were randomly divided into 2 groups (Conventional band and loop space maintainer (C-BLSM) and 3D-printed band and loop space maintainer (3D-BLSM)). The randomization of the groups was done using computer-generated randomization (https://www.random.org) by a statistician to ensure unbiased group allocation (Fig. 1).

### Group I: conventional band and loop space maintainer (C-BLSM)

The smallest stainless steel band (3M, Minnesota, USA) that fit was chosen for each C-BLSM. Impressions were taken with alginate material with a drop of super glue in the impression substance to stabilize the band. Within 30 min of taking the impression, the impression was poured using a dental stone. The loops were made using 0.036-inch steel wires and flux (Dentaurum Dentaflux Universal Dentaflux®, New Delhi, India). The same technician worked at the laboratory for all of the research space maintainers. Seven days later, the children were scheduled for space maintenance and cementation. Cotton roll isolation was utilized, and cementation was done with GC Fuji (Tokyo, Japan). The participants were not allowed to eat for 30 min after cementation. Parents were given post-cementation care instructions and asked to call if they had any issues. Follow-up appointments were planned at 1, 3, 6, and 9 months after the cementation.

### Group II: three-dimensionally (3D) printed band and loop space maintainer (3D-BLSM)

**Figure 2:**
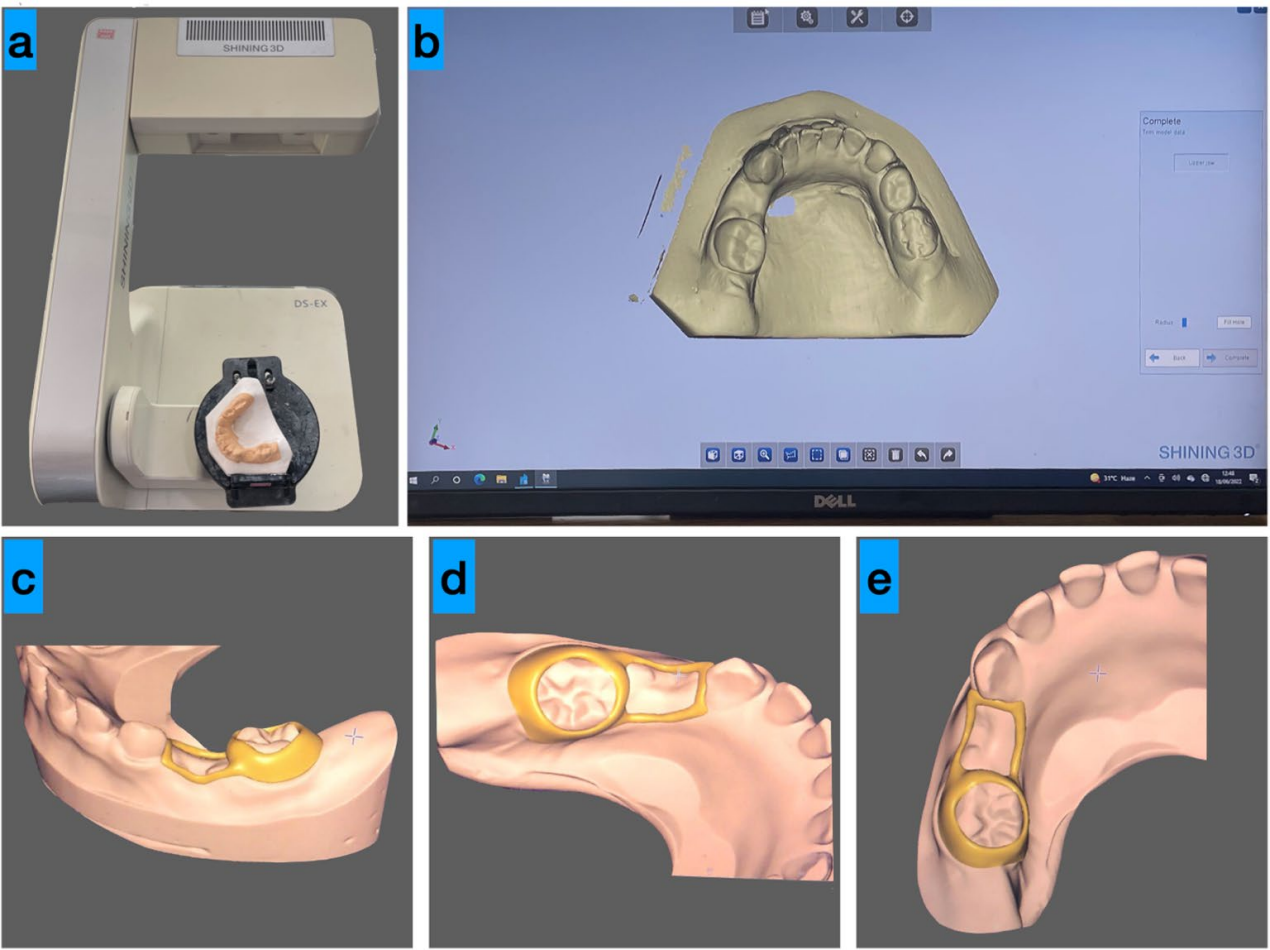
Manufacturing process of 3D-BLSM. (**a**) Scanning device for scanning the retrieved cast; (**b**) scanned cast digital model; and (**c–e**) the digital design of a BLSM similar to conventional.

A single-step rubber-based impression with the addition of silicon was made, and a cast was poured and retrieved. A 3D digital dental scanner (AutoScan-DS-EX—Shining 3D, Zhejiang, China) was used to scan the cast (Fig. 2a,b). DentalCAD 2.2 Valletta software (Exocad GmbH, Darmstadt, Germany) was then used to create the band and loop, which mirrored the traditional space maintainer (Fig. 2c–e). The titanium-based powdered metal material (Ti64 Gd23; LPW Technology Ltd., Cheshire, UK) used for fabricating the 3D-designed space maintainer was subsequently generated using micro laser sintering technology, which incorporates the benefits of additive manufacturing techniques. Following that, the 3D-BLSM was carefully placed into the patient’s mouth cavity to guarantee a precise fit. After the patient confirmed a satisfactory fit, the 3D-BLSM was securely bonded in place with glass ionomer cement (Type 2; GC Fuji; Tokyo, Japan). Following the procedure, the patient was advised not to eat food or beverages for 30 min and not to bite on hard objects to allow the cement to fully cure. Parents were given post-cementation care instructions and instructed to contact the office if they had any problems. Follow-up visits were scheduled in a similar way as described in group I.

**Figure 3:**
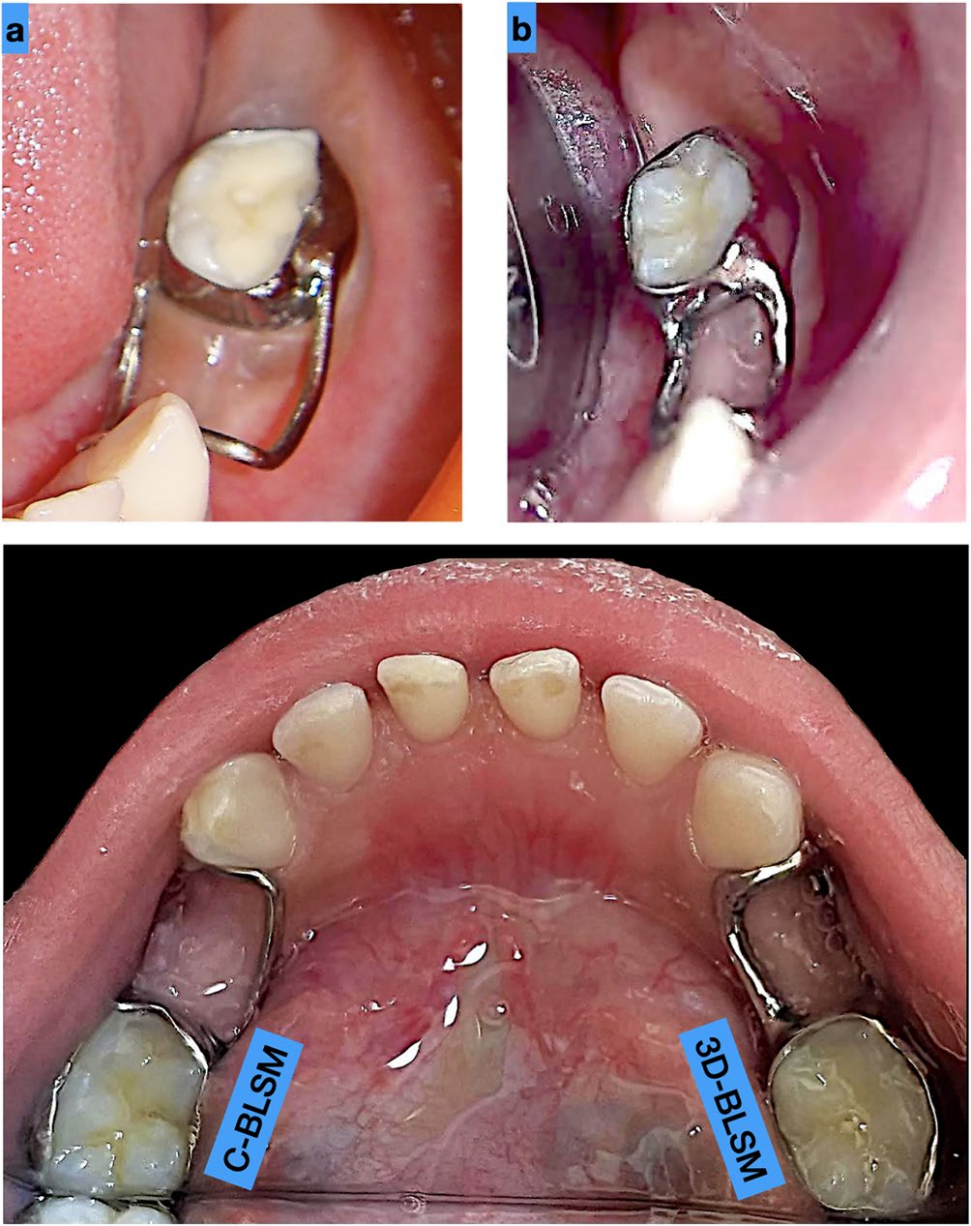
Cementation of space maintainers. (**a**) C-BLSM; (**b**) 3D-BLSM; and (**c**) both types of space maintainers in a single patient.

The placements of the two experimental space maintainers are presented in Fig. 3a,b. Also, during this study we found a case a bilateral requirement of space maintainers but was not a part of the study. The case representation is reported it in Fig. 3c.

### Evaluation criteria of the space maintainers

#### Assessment of space maintainer failure prevalence

##### Survival time of SMs

Potential concerns like as de-cementation, debonding, solder breakage, loop breakage, band breakage, and abutment tooth fracture were evaluated for both space maintainers.

##### Gingival health of the abutment tooth

The gingival state of both abutment teeth was evaluated using the criteria established by Löe and Silness in 1963^20^ (Table 1), and the results were documented in each follow-up appointment.

**Table 1:** Score of the health of gingiva according to Löe and Silness.

Scores	Status of the gingiva	Clinical features
0	Normal	Natural coral pink gingiva with no evidence of inflammation
1	Mild Inflammation	Slight changes in colour, slight oedema No bleeding on probing
2	Moderate Inflammation	Redness, edema and glazing Bleeding upon probing
3	Severe Inflammation	Marked redness and oedema ulceration/tendency to bleed spontaneously

#### Patients’ satisfaction

The overall satisfaction of patients was assessed using a 5-point Likert-type scale 2, and the findings were collected in each follow-up appointment. To measure the satisfaction of both parents and patients, a 5-point Likert-type scale was used^21^. Parents were asked to rate the form, durability, general pleasure, and comfort of using the space maintainer. In addition, children were asked to rate their overall experience including potential concerns.

A pilot study was conducted before the main study for examiner calibration and to assess the intra-examiner and inter-examiner reliability. Two examiners participated (D.A.W. and A.M.L.) in the pilot study along with 10 participants, and the intra-class correlation coefficient (ICC) was assessed. The principal examiner observed the outcome of the 10 participants twice (within a three-day interval). The intra-class correlation coefficients for intra-examiner reliability were between 0.966 and 0.986. As a result, there was significant intra-examiner repeatability (ICC > 0.750). Also, the inter-examiner reliability ICC ranged from 0.896 to 0.984.

### Statistical analysis

The data were collected and documented in Microsoft Excel version 13. Following that, IBM Statistical Packages for Social Sciences (SPSS) Version 21 was employed for statistical analysis. The frequency and percentage distributions of categorical data were computed. The Kruskal–Wallis test was used to compare the gingival index and Likert scale addresses across periods. In addition, the Mann–Whitney U test was used to compare C-BLSM with 3D BLSM. The Kaplan–Meier Test was used for survival analysis. Throughout all analyses, a 95% confidence interval was maintained, and statistical significance was set at a p-value of < 0.05.

## Results

### Survival

A total of 40 (64.5%) space maintainers survived among the 62 participants (100%). C-BLSM had 21 (67.7%) survivals at 3 months after treatment, while 3D-BLSM had 27 (87.1%) survivals; however, this difference in proportion was not statistically significant (p > 0.05). The 6-month analysis found that 17 (54.8%) of C-BLSM patients survived, and so did 26 (83.9%) of 3D-BLSM patients with a statistically significant difference in proportion (p < 0.05). Furthermore, after 9 months, C-BLSM had 16 (51.6%) survivals, but 3D-BLSM had 24 (77.4%) with a statistically significant difference in proportions (p < 0.05) (Table 2).

**Table 2:** The survival of C-BLSM and 3D-BLSM at different time intervals.

			Status		Total	p value
			Failure	Survived		
Baseline	C-BLSM	n	0	31	31	–
		%	0	100.0%	100.0%	
	3D-BLSM	n	0	31	31	
		%	0	100.0%	100.0%	
Total		n	0	62	62	
		%	0	100.0%	100.0%	
3 months	C-BLSM	n	10	21	31	0.068
		%	32.3%	67.7%	100.0%	
	3D-BLSM	n	4	27	31	
		%	12.9%	87.1%	100.0%	
Total		n	14	48	62	
		%	22.6%	77.4%	100.0%	
6 months	C-BLSM	n	14	17	31	0.013
		%	45.2%	54.8%	100.0%	
	3D-BLSM	n	5	26	31	
		%	16.1%	83.9%	100.0%	
Total		n	19	43	62	
		%	30.6%	69.4%	100.0%	
9 months	C-BLSM	n	15	16	31	0.034*
		%	48.4%	51.6%	100.0%	
	3D-BLSM	n	7	24	31	
		%	22.6%	77.4%	100.0%	
Total		n	22	40	62	
		%	35.5%	64.5%	100.0%	

*Statistically significant at p < 0.05.

### Survival rate

**Figure 4:**
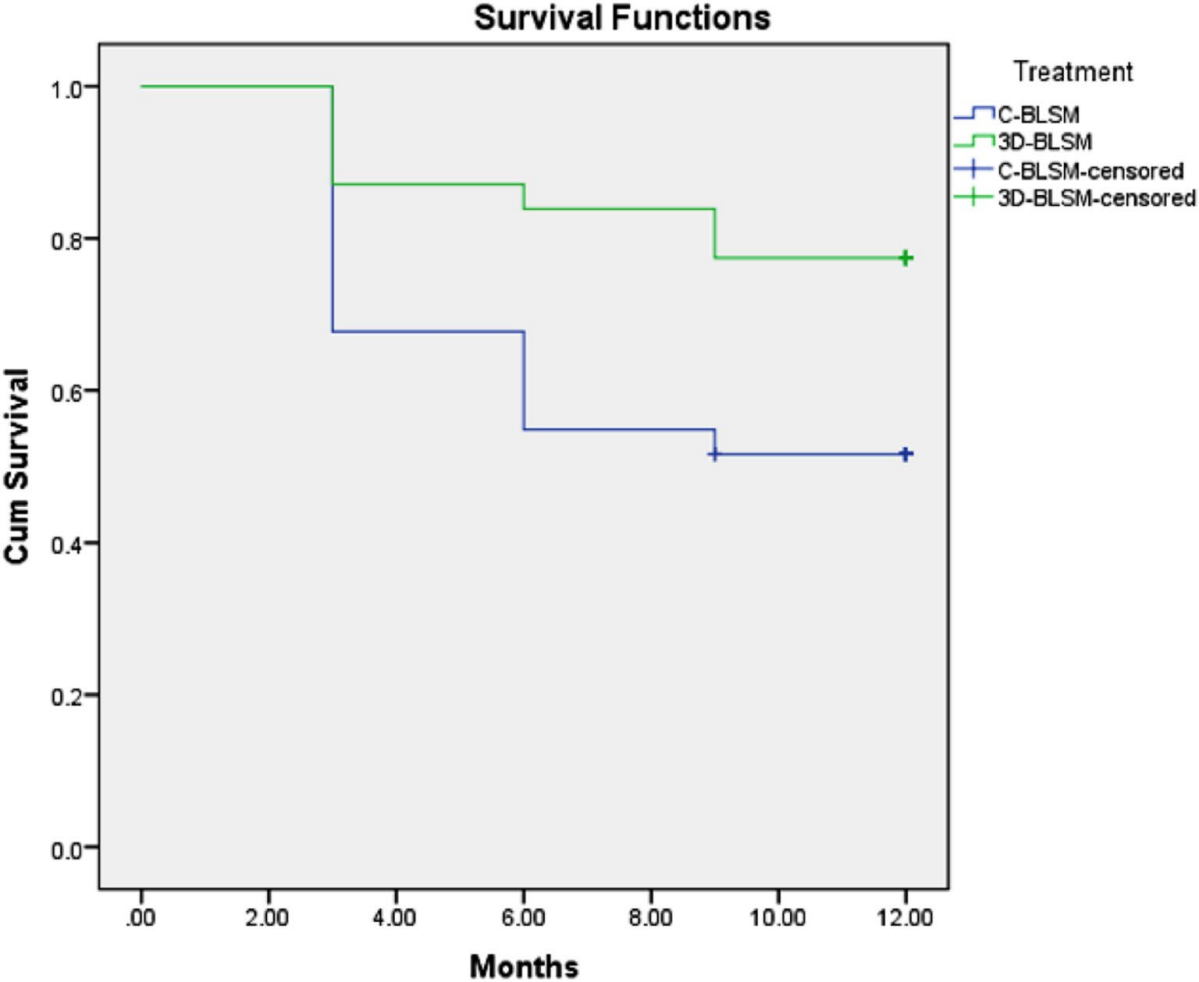
Survival analysis of the C-BLSM and 3D-BLSM.

The mean survival rate in the C-BLSM Group was estimated to be 8.22 ± 0.737 months. Whereas, in the 3D BLSM Group, the mean survival rate was estimated to be 10.45 ± 0.562 months. This difference in proportion was found to be statistically significant (p < 0.05) (Table 3). The survival analysis is presented in Fig. 4.

**Table 3:** Mean survival rate in C-BLSM and 3D BLSM.

	Estimate	Std. error	95% confidence interval		p value
			Lower bound	Upper bound	
C-BLSM	8.226	0.737	6.782	9.670	0.030*
3D-BLSM	10.452	0.562	9.350	11.553	
Overall	9.339	0.484	8.389	10.288	

*Statistically significant at p < 0.05.

### Gingival health

The gingival index was evaluated within the C-BLSM group, and a statistically significant increase in the percentage of patients displaying moderate inflammation was seen from the baseline to 9 months, ranging from 0 to 15.30% (p < 0.05). Similarly, in the 3D BLSM group, the gingival index increased significantly from the baseline to nine months in people with moderate inflammation, going from 0 to 7.30% (p < 0.05) (Table 4).

**Table 4:** Comparison of the gingival index in both space maintainers evaluated at different time intervals.

			GROUPS		Total	p value
			C-BLSM	3D-BLSM		
Baseline	Normal	N	17	22	39	0.19
		%	27.40%	35.50%	62.90%	
	Mild inflammation	N	14	9	23	
		%	22.60%	14.50%	37.10%	
3 Months	Normal	N	5	8	13	0.30
		%	8.10%	12.90%	21.00%	
	Mild inflammation	N	20	19	39	
		%	32.30%	30.60%	62.90%	
	Moderate inflammation	N	6	4	10	
		%	9.70%	6.50%	16.10%	
6 Months	Mild inflammation	N	25	25	50	1.00
		%	40.30%	40.30%	80.60%	
	Moderate inflammation	N	6	6	12	
		%	9.70%	9.70%	19.40%	
9 Months	Normal	N	2	2	4	0.022*
		%	3.20%	3.20%	6.50%	
	Mild inflammation	N	10	20	30	
		%	16.10%	32.30%	48.40%	
	Moderate inflammation	N	19	9	28	
		%	30.60%	14.50%	45.20%	
Total		N	31	31	62	
		%	50.00%	50.00%	100.00%	

*Statistically significant at p < 0.05.

When the gingival index was compared from the baseline to 9 months, a higher percentage of participants which were 22 (35.50%) in the 3D-BLSM Group were found at the baseline. However, this difference between the groups at the baseline was not statistically significant (p > 0.05). After 3 months, a higher proportion of participants, 20 participants in the C-BLSM Group, reported having mild inflammation (32.30%). After 6 months, the proportion of participants in both groups with mild and moderate inflammation was equal. It means that this difference in proportion was not statistically significant (p > 0.05). After 9 months, a higher proportion of participants with moderate inflammation of the gingiva came from the C-BLSM Group (p < 0.05) (Table 4).

### Patients’ overall satisfaction

The Likert-scale assessment in the C-BLSM Group from the baseline to 9 months depicted 36 participants with neutral opinions (29%), one of which (0.80%) at the baseline reported neutral C-BLSM after 3 months. After 3 months, the proportion of participants with neutral opinions increased to 7 (5.6%). The proportion after 6 months doubled to 14 (11.30%), and the same proportion (11.30%) was found after 9 months. These differences in proportion were statistically significant (p < 0.05).

Results in the 3D-BLSM Group from the baseline to 9 months showed a higher proportion of participants with opinions of Agree (49; 39.50%). Nine of them (7.30%) at the baseline reported agreement on 3D-BLSM after 3 months. The proportion remained constant at 9 (7.30%) after 6 months. The proportion of participants who agreed was 18 (14.5%). After 9 months, the proportion of participants who agreed decreased to 13 (10.50%). These differences in proportion were statistically significant (p < 0.05).

When the Likert-scale results were analyzed from the baseline (1-month), 3-month, 6-month, and 9-month evaluation, it was observed that the difference in the proportion of responses was statistically significant. After 6 months wherein 9 participants (14.50%) of the C-BLSM Group strongly disagreed. Meanwhile, 18 participants of the 3D BLSM Group agreed (29%). These differences in proportion were observed to be statistically significant (p < 0.05) (Table 5).

**Table 5:** Comparison of the Likert-scale responses with respect to overall satisfaction of patients’ in C-BLSM and 3D-BLSM groups at different time intervals.

			GROUPS		Total	p value
			C-BLSM	3D-BLSM		
Baseline	Strongly disagree	N	5	7	12	0.080
		%	8.10%	11.30%	19.40%	
	Disagree	N	4	4	8	
		%	6.50%	6.50%	12.90%	
	Neutral	N	1	3	4	
		%	1.60%	4.80%	6.50%	
	Agree	N	4	9	13	
		%	6.50%	14.50%	21.00%	
	Strongly agree	N	17	8	25	
		%	27.40%	12.90%	40.30%	
3 Months	Strongly disagree	N	0	4	4	0.780
		%	0.00%	6.50%	6.50%	
	Disagree	N	7	3	10	
		%	11.30%	4.80%	16.10%	
	Neutral	N	7	5	12	
		%	11.30%	8.10%	19.40%	
	Agree	N	5	9	14	
		%	8.10%	14.50%	22.60%	
	Strongly agree	N	12	10	22	
		%	19.40%	16.10%	35.50%	
6 Months	Strongly disagree	N	9	5	14	0.000*
		%	14.50%	8.10%	22.60%	
	Disagree	N	5	0	5	
		%	8.10%	0.00%	8.10%	
	Neutral	N	14	5	19	
		%	22.60%	8.10%	30.60%	
	Agree	N	2	18	20	
		%	3.20%	29.00%	32.30%	
	Strongly agree	N	1	3	4	
		%	1.60%	4.80%	6.50%	
9 Months	Strongly disagree	N	0	4	4	0.260
		%	0.00%	6.50%	6.50%	
	Disagree	N	0	1	1	
		%	0.00%	1.60%	1.60%	
	Neutral	N	14	4	18	
		%	22.60%	6.50%	29.00%	
	Agree	N	13	13	26	
		%	21.00%	21.00%	41.90%	
	Strongly agree	N	4	9	13	
		%	6.50%	14.50%	21.00%	
Total		N	31	31	62	
		%	50.00%	50.00%	100.00%	

*Statistically significant at p < 0.05.

## Discussion

Space maintainers are essential in pediatric dentistry because they help preserve the correct spacing among the dental arch resulting from the premature loss of primary teeth, thereby avoiding unwanted dental movements and misalignments. The importance of space maintainers becomes apparent when they prevent malocclusions and orthodontic issues that might occur due to premature loss of primary teeth. This preventive strategy is especially important during the transitional period between primary and permanent dentition as it lays the foundation for appropriate occlusion and alignment^22^.

Recent advancements in space maintenance technology, like as the use of 3D printing, have rendered previous approaches obsolete. 3D printing creates accurate and patient-specific equipment, allowing for bespoke solutions for particular dental problems while also improving accuracy and efficiency. This technological integration is consistent with the larger trend of digital dentistry, which involves the use of digital impressions and computer-aided design (CAD) for precise mapping of the oral environment, resulting in highly personalized space maintainers that not only improve effectiveness but also promote patient comfort and acceptance^23^.

Furthermore, the recent incorporation of advanced materials in 3D printing for space maintainers, such as biocompatible and durable resins, contributes to the evolution of dental interventions. Those advancements address limitations associated with traditional materials and ensure improved strength and longevity. They represent a progressive shift toward more customized, precise, and technologically advanced approaches to space maintenance in pediatric dentistry, ultimately improving the standard of care and treatment outcomes for young patients during critical periods of dental development^15,16,23,24^.

The therapeutic efficacy and survival time of several forms of space maintainers (SMs) have been investigated by researchers. SMs have a broad range of effectiveness rates, ranging from 92 to 27%, according to previous studies^25–27^. The effectiveness of 3D-BLSM has not been widely studied. Band and loop SMs (BL SMs) are often used for conserving space after early loss of primary teeth due to their historically high success rates over long durations^18,27,28^. As a result, the current findings shed light on the comparison between C-BLSM and the most contemporary 3D-BLSM, evaluating its clinical performance and influence on oral hygiene.

The success rate of C-BLSM was determined to be 92% as Tahririan et al.^25^ reported in their study. Other trials found similar success rates, such as 90%^27^, 86.7%^26^, and 84.6%^29^. In a long-term investigation, Sasa et al.^30^, observed a much lower success rate of 42.5%. The survival rate for C-BLSM in the current study at the end of the 9-month evaluation was 51.6%.

In the current study, significant variations are put forward over time when contrasting C-BLSM to 3D-BLSM. While the 3-month survival rates from both types of maintainers were comparable (87.1% for 3D-BLSM vs. 67.7% for C-BLSM). The evaluations indicated considerably higher survival rates for 3D-BLSM (83.9% after 6 months and 77.4% after 9 months) compared to C-BLSM (54.8% after 6 months and 51.6% after 9 months). These findings indicate 3D-BLSM’s greater long-term durability in retaining space within the dental arch as compared to regular C-BLSM. The survival rate of space maintainers is critical since it is directly related to the efficacy and success of the execution^31^.

The substantially increased survival rate observed among 3D-BLSM in our study can be credited to 3D printing technology’s distinct benefits. 3D printing’s personalization enables exact adaptation to unique patient anatomy, potentially minimizing soft tissue pain and interference with adjoining teeth. Furthermore, 3D printing material properties like as enhanced durability and biocompatibility eventually result in a stronger device that is less susceptible to fractures. The fabrication precision of 3D printing may result in less plaque retention on smoother surfaces, minimizing the risk of dental cavities and gingival discomfort. Patient-specific design components, made feasible by 3D printing, improve appliance operation, and sophisticated layer-by-layer production procedures lead to a structurally better device. Furthermore, the better biocompatibility of 3D printing materials encourages a more favorable tissue response, minimizing the risk of soft tissue irritation and boosting overall patient comfort and acceptance^32–35^.

The current research findings have important therapeutic significance in pediatric dentistry, providing insight into the effectiveness of conventional band and loop space maintainers in comparison to their 3D-printed equivalents. Dentists may now customize treatment plans based on patient-specific criteria, taking into account the benefits and downsides of each type of space maintainer. This evidence-based decision-making is critical for selecting a space maintainer after the loss of primary teeth, thereby improving patient care quality. Furthermore, the study’s examination of patient comfort and acceptability associated with both traditional and 3D-printed space maintainers emphasizes the importance of a patient-centered approach in treatment planning to inquire about the incorporation of innovative technologies into clinical practice.

Because meticulous clinical trials on 3D-BLSM have not yet been conducted, a direct comparison of the success rates identified in our investigation with the available literature is not possible. Particularly, Pawar^18^ provided the first academic paper on this kind of SM as a case report on a 7-year-old kid with no detected constraints during clinical observations at the 3-month follow-up. Following the case report by Khanna et al.^19^, additional short-term follow-ups were provided to attend justifiable results. Considering the least number of literature on the effectiveness and long-term efficacy of 3D-BLSM, more extensive clinical studies are required.

In our study, a substantial difference in the impact of SMs on oral hygiene was observed between the two groups. Importantly, regardless of the type of fixed SM, our data revealed a rise in the abutment tooth’s gingival index (GI) values, consistent with the findings of Arikan et al.^36^ who discovered that GI levels increased considerably in the first and third months when compared to original data. Similarly, Hosseinipour et al.’s^37^ study on the influence of fixed SMs on oral hygiene showed a significant increase in GI scores after six months of treatment. Contrastly, our study found that patients’ general GI scores decreased, possibly due to the Hawthorne effect^38^, in which participants adjust their behavior when they are aware that they are being watched. Oral hygiene stimulus during control check-ups, using plaque-revealing technologies, can be employed to offset negative impacts on the periodontal health of abutment teeth^39^.

Remarkably, based on the Likert-scale responses, a change in patient opinion was found at the 6-month follow-up. In contrast, the 3D BLSM group showed a statistically significant increase (29%) in “agree” responses, but the C-BLSM group showed a statistically significant rise (14.50%) in “strongly disagree” responses. This discrepancy suggests a brief decline in C-BLSM satisfaction at this intermediate stage which needs to be further investigated. Interestingly, after nine months, this difference disappeared, supporting the results of Rana et al., while results during this period indicated that both modalities provide similar long-term happiness. The 3D BLSM’s ability to provide a respectable substitute for the well-established C-BLSM approach is intriguing^11^.

The current findings can help dentists customize treatments based on patient preferences to improve treatment compliance and outcomes. In resource-constrained environments, decision-making should be guided by an assessment of the economic consequences of traditional and 3D-printed space maintainers. Education programs need to be promoted to practitioners to integrate innovative technology such as 3D printing in dentistry. Furthermore, the current findings help long-term treatment planning in pediatric dentistry, allowing for methods that can result in both well-performed immediate and long-term outcomes.

This current study promotes interdisciplinary teamwork, especially among patients who have several oral problems. It emphasizes the importance of evidence-based, patient-centered decision-making in enhancing the quality of treatment for young children who have lost their primary teeth. While the study’s hypotheses are consistent with the observed results, further research, particularly the biomechanical one, is required to completely understand the underlying processes from the improved survival rate of 3D-BLSM revealed in our study.

This current study had a potentially limited sample size, which impacts generalizability and thus needs bigger and more varied cohorts in future research. The lack of biomechanical evaluations indicates the need for further investigation of mechanical variables that influence device performance. Variations in operator expertise in manufacture and placement might add confounding effects, prompting attention in future studies. Moreover, longitudinal studies in pediatric dentistry should be conducted in the future to determine the long-term efficacy and survival rates of 3D-printed space maintainers. Biomechanical studies that investigate structural characteristics and forces involved can provide insights into device performance. Comparative studies with a larger and more varied patient population will improve external validity, and investigating patient-reported outcomes like comfort and satisfaction will lead to a more comprehensive knowledge of the space maintainer’s function. Furthermore, healthcare decision-makers and practitioners must investigate the cost-effectiveness and economic consequences of both types of space maintainers.

Besides sample size, this study does not address the variability of operator experience in production and placement, which may affect aspects like as comfort and fit. Therefore, a more diversified cohort is necessary for thorough insights. Limitations found in this study are expected to be addressed in future research.

## Conclusion

In pediatric dentistry, this study clearly illustrates the extent to which 3D-printed space maintainers (3D-BLSMs) function compared to their traditional equivalents, such as band and loop space maintainers (C-BLSMs). 3D-BLSMs showed an impressive 77.4% retention rate over 9 months with a notable 25.8% improvement above the retention seen in C-BLSMs (51.6%). This stark distinction highlights the improved long-term durability that 3D printing technology can offer. Patient satisfaction was substantial in both groups even though using both space maintainer types caused a statistically significant increase in the gingival index, highlighting the critical importance of strict oral hygiene habits. These intriguing findings support the deliberate adoption of 3D-BLSMs as an additional persistent and dependable alternative within the clinical armamentarium, which might pave the way for improved treatment outcomes and patient satisfaction in pediatric dentistry.

### Supplementary Information

Supplementary Information 1.

Supplementary Information 2.

## Data Availability

The data related to the study have been uploaded as Supplementary Material.
