# Supplementary material for: Effectiveness of traditional band and loop space maintainer vs 3D-printed space maintainer following the loss of primary teeth: a randomized clinical trial
Source: Sci Rep. 2024 Jun 18;14:14081. doi: 10.1038/s41598-024-61743-7 (PMC11189383; doi:10.1038/s41598-024-61743-7)
Supplement: Supplementary file 2 — Supplementary Information 2. [file 41598_2024_61743_MOESM2_ESM.pdf]

Clinical Trial Details (PDF Generation Date :- Fri, 30 Jun 2023 04:26:55 GMT)

**CTRI Number**	CTRI/2023/06/054553 [Registered on: 30/06/2023] - **Trial Registered Prospectively**
**Last Modified On**	29/06/2023
**Post Graduate Thesis**	No
**Type of Trial**	Interventional
**Type of Study**	Dentistry
**Study Design**	Randomized, Parallel Group Trial
**Public Title of Study**	3D printed space maintainer (3D-SM) vs. traditional band and loop space maintainer (C-BLSM)
**Scientific Title of Study**	Effectiveness of 3D printed space maintainer Vs traditional Bond and loop space maintainer following loss of primary teeth. A randomized clinical trial.

Secondary IDs if Any

Secondary ID	Identifier
NIL	NIL

Details of Principal Investigator or overall Trial Coordinator (multi-center study)

**Details of Principal Investigator**	
**Name**	Anuj Bhardwaj
**Designation**	Professor and Head
**Affiliation**	College of Dental Sciences and Hospital
**Address**	Department of Conservative Dentistry, 301, 1st Floor, Jhoomar Ghat AB Rd near Hotel Mashal Rau Madhya Pradesh<br>Indore<br>MADHYA PRADESH<br>453331<br>India
**Phone**	
**Fax**	
**Email**	dranuj_84@yahoo.co.in

Details Contact Person (Scientific Query)

**Details Contact Person (Scientific Query)**	
**Name**	Anuj Bhardwaj
**Designation**	Professor and Head
**Affiliation**	College of Dental Sciences and Hospital
**Address**	Department of Conservative Dentistry, 301, 1st Floor, Jhoomar Ghat AB Rd near Hotel Mashal Rau Madhya Pradesh<br>Indore<br>MADHYA PRADESH<br>453331<br>India
**Phone**	
**Fax**	
**Email**	dranuj_84@yahoo.co.in

Details Contact Person (Public Query)

**Details Contact Person (Public Query)**	
**Name**	Anuj Bhardwaj
**Designation**	Professor and Head
**Affiliation**	College of Dental Sciences and Hospital
**Address**	Department of Conservative Dentistry, 301, 1st Floor, Jhoomar Ghat AB Rd near Hotel Mashal Rau Madhya Pradesh<br>Indore<br>MADHYA PRADESH<br>453331<br>India
**Phone**	

**Fax**	
**Email**	dranuj_84@yahoo.co.in

Source of Monetary or Material Support

Source of Monetary or Material Support
> College of Dental Sciences and Hospital Rau Indore

Primary Sponsor

Primary Sponsor Details	
**Name**	Dr Anuj Bhardwaj
**Address**	College Of Dental Science and Hospital F12 Jhoomar Ghat AB Rd near Hotel Mashal Rau Madhya Pradesh 453331
**Type of Sponsor**	Other [Self]

Details of Secondary Sponsor

Name	Address
NIL	NIL

Countries of Recruitment

List of Countries
India

Sites of Study

Name of Principal Investigator	Name of Site	Site Address	Phone/Fax/Email
Dr Anuj Bhardwaj	College Of Dental Science and Hospital	301, third floor, Department of Conservative Dentistry and Endodontics, F12 Jhoomar Ghat AB Rd near Hotel Mashal Rau Madhya Pradesh 453331<br>Indore<br>MADHYA PRADESH	7898540222<br><br>dranuj_84@yahoo.co.in

Details of Ethics Committee

Name of Committee	Approval Status	Date of Approval	Is Independent Ethics Committee?
Institutional Ethics Committee of College Of Dental Science & Hospital	Approved	19/01/2023	No

Regulatory Clearance Status from DCGI

Status	Date
Not Applicable	No Date Specified

Health Condition / Problems Studied

Health Type	Condition
Patients	Disturbances in tooth eruption

Intervention / Comparator Agent

Type	Name	Details
Intervention	3D Printed Space Maintainer	The 3D printed space maintainer (3D-SM) is a novel device that can be custom-designed and fabricated in a single visit. The placement will be done and will be evaluated till the eruption of the permanent tooth (6-12 months).
Comparator Agent	Conventional Band and loop space maintainer	The most used SMs are the conventional band and loop space maintainers (C-BLSM), which are fabricated in a dental laboratory and require multiple visits for placement. The placement will be done and will be evaluated till the eruption of

		the permanent tooth (6-12 months).

Inclusion Criteria

Inclusion Criteria	
**Age From**	4.00 Year(s)
**Age To**	8.00 Year(s)
**Gender**	Both
**Details**	Inclusion Criteria<br>Clinical criteria<br>Systemically healthy children.<br>Grossly mutilated single molars on either side, requiring<br>extraction.<br>Freshly extracted single molars bilaterally in the same arch<br>or opposite arch.<br>Sound and healthy teeth adjacent to the extraction site.<br>No abnormal dental conditions such as crossbite, open bite,<br>and deep bite.<br>Radiographic criteria<br>Presence of succedaneous tooth bud.<br>Presence of at least 1 mm bone overlying the succedaneous<br>tooth germ with less than one-third of the root formed.

Exclusion Criteria

Exclusion Criteria	
**Details**	Grossly carious teeth were adjacent to the created space. Absence of teeth on the mesial or distal side of the teeth to be extracted.

**Method of Generating Random Sequence**	Computer generated randomization
**Method of Concealment**	Centralized
**Blinding/Masking**	Outcome Assessor Blinded

Primary Outcome

Outcome	Timepoints
Survival Time of Space maintainers compared Gingival Health of the Abutment Tooth	follow up intervals 0 1 3 6 and 9 months

Secondary Outcome

Outcome	Timepoints
Patient Satisfaction	follow up intervals 0 1 3 6 and 9 months

**Target Sample Size**	**Total Sample Size=**30<br>**Sample Size from India=**30<br>**Final Enrollment numbers achieved (Total)=**Applicable only for Completed/Terminated trials<br>**Final Enrollment numbers achieved (India)=**Applicable only for Completed/Terminated trials
**Phase of Trial**	Phase 2
**Date of First Enrollment (India)**	07/07/2023
**Date of First Enrollment (Global)**	No Date Specified
**Estimated Duration of Trial**	**Years=**0<br>**Months=**10<br>**Days=**0
**Recruitment Status of Trial (Global)**	Not Applicable
**Recruitment Status of Trial (India)**	Not Yet Recruiting
**Publication Details**	
**Brief Summary**	The premature loss of primary teeth can lead to several dental problems, including space loss, malocclusion, and impacted permanent teeth. Space maintainers (SMs) are dental devices used to preserve space and prevent these problems from occurring. The most used SMs are the conventional band and loop space maintainers (C-BLSM), which are fabricated in a dental laboratory and require multiple visits for placement.

Recent advancements in technology have led to the development of 3D printing in dentistry, which has the potential to revolutionize the fabrication of SMs. The 3D printed space maintainer (3D-SM) is a novel device that can be custom-designed and fabricated in a single visit. However, there is limited research on the effectiveness of 3D-SMs compared to traditional C-BLSMs.

Therefore, this randomized clinical trial aims to compare the effectiveness of 3D-SMs and C-BLSMs in preserving space and preventing dental problems following the loss of primary teeth. The trial will assess the survival rate, clinical performance, and patient satisfaction of both types of SMs. The study will also evaluate the time and cost required for the fabrication and placement of each type of SM. The findings of this study will provide valuable insights into the effectiveness of 3D-SMs compared to traditional C-BLSMs and their potential for use in clinical practice. This research will also contribute to the growing body of literature on the application of 3D printing technology in dentistry and its potential to improve patient outcomes.
